# Short-Term COVID-19 Pandemic-Related Endoscopy Delays Did Not Translate to Deleterious Outcomes for Patients With Inflammatory Bowel Disease: A Retrospective Cohort Study

**DOI:** 10.1093/jcag/gwac009

**Published:** 2022-03-16

**Authors:** Jason Chambers, Gurpreet Malhi, Maria Mikail, Reena Khanna, Aze Wilson

**Affiliations:** Schulich School of Medicine and Dentistry, Western University, 1151 Richmond St, London, Ontario N6A 5C1, Canada; Schulich School of Medicine and Dentistry, Western University, 1151 Richmond St, London, Ontario N6A 5C1, Canada; Schulich School of Medicine and Dentistry, Western University, 1151 Richmond St, London, Ontario N6A 5C1, Canada; Department of Medicine, Division of Gastroenterology, Western University, London, Ontario, Canada; Department of Medicine, Division of Gastroenterology, Western University, London, Ontario, Canada; Department of Medicine, Division of Clinical Pharmacology, Western University, London, Ontario, Canada; Department of Physiology and Pharmacology, Western University, London, Ontario, Canada

**Keywords:** Inflammatory bowel disease, COVID-19 pandemic, Endoscopy delays, Surgery, Hospitalization, ER access

## Abstract

**Background:**

The management of inflammatory bowel disease (IBD) requires frequent endoscopic assessment. It is unknown if measures put in place to reduce the spread of the virus SARS-CoV-2, including the delay of non-urgent patient assessments, resulted in deleterious outcomes for patients with IBD. Therefore, we aimed to determine if delays in endoscopy during the COVID-19 pandemic were associated with an increased risk of adverse IBD outcomes (emergency room, ER presentation, hospitalization, surgery, or escalation of drug therapy).

**Methods:**

A retrospective cohort study was performed in patients with IBD scheduled for outpatient endoscopies between March and August 2019 and 2020 at two tertiary care centers affiliated with Western University, London, Canada. Data pertaining to endoscopy timing, IBD drug prescription, ER attendance, hospitalization, and surgery were collected.

**Results:**

A total of 1160 endoscopies (2019, *n* = 718; 2020, *n* = 442) occurred during the study periods in 669 (2019) and 414 (2020) patients with IBD, respectively. More endoscopies were delayed in 2020 than 2019 (26.7% vs. 9.7%, respectively, *P* < 0.0001). Endoscopy delay was not associated with an increased risk of an adverse IBD outcome (OR = 1.23, 95%CI = 0.89–1.34, *P* = 0.20). Fewer adverse IBD outcomes were seen in the 2020 cohort who had endoscopy delays (*n* = 33/115, 28.7%) versus those without delay (*n* = 176/299, 58.9%, *P* < 0.0001).

**Conclusion:**

More endoscopy delays occurred during the COVID-19 pandemic; however, delays in general were not associated with adverse IBD outcomes, and in particular, endoscopy delays during the COVID-19 pandemic were associated with fewer deleterious IBD outcomes, suggesting that patients with IBD in need of urgent endoscopy were appropriately identified.

## INTRODUCTION

Inflammatory bowel disease (IBD) is an autoimmune disease characterized by a chronic relapsing-remitting pattern of inflammation within the gastrointestinal tract (GI) and includes two forms: Crohn’s Disease (CD) and ulcerative colitis (UC) (1). Due to the chronicity of IBD and the risk of deleterious outcomes such as hospitalization, malnutrition and intestinal resection, objective monitoring of intestinal disease activity and aggressive control of the disease early in its course are vital for improving patient outcomes and reducing healthcare costs (2–5). A mainstay of disease assessment and surveillance is endoscopy, which includes esophagogastroduodenoscopy (EGD) and ileocolonoscopy (6). Direct visualization of the affected areas throughout the GI tract and the collection of biopsy samples allow clinicians to adjust the application of medical therapy and maximize drug efficacy and minimize adverse disease outcomes (3,4). Given this, individuals with IBD regularly undergo endoscopic assessment and any delay in this procedure may have important ramifications for disease-related outcomes and effective disease management.

In March 2020, the coronavirus disease (COVID-19) caused by the SARS-CoV-2 virus was declared a global pandemic by the World Health Organization (WHO) (7). With this declaration came the rapid implementation of new infection control measures across health care systems. Minimizing contact between health care providers and patients was a key part of new infection control mandates. For individuals with IBD, there was a shift from in-person appointments to telephone or virtual assessments and a call to reduce access to non-urgent endoscopy (8,9). The extent of COVID-19-related delays in endoscopy and their impact on IBD-related outcomes are unknown. We, therefore, aimed to evaluate the length and number of endoscopy delays during the COVID-19 pandemic for individuals with IBD and the association with major adverse IBD events.

## METHODS

### Study Design and Participants

A retrospective cohort study was conducted in individuals with IBD (either UC or CD) presenting for an outpatient endoscopic procedure at two tertiary care centres affiliated with Western University, London, Canada and seen between March 1, 2019 and August 31, 2019 (2019 cohort, pre-COVID-19 pandemic) and March 1, 2020 and August 31, 2020 (2020 cohort, during COVID-19 pandemic). Participants were required to be 18 years of age or older and have a confirmed diagnosis of one of UC or CD based on the combination of endoscopic, radiographic and histological features. All IBD diagnoses were made prior to the study period of March 1, 2019. Baseline data were collected on all participants including age, sex, disease type (CD or UC) and rurality based on postal code where postal codes containing ‘0’ as the second character are deemed rural based on the Canada Post Corporation, endoscopy type, scheduled endoscopy date, actual endoscopy date and reason for endoscopy. Other data collected included were emergency room (ER) attendance, need for surgical resection, hospitalization and escalation of IBD drug therapy. Individuals were excluded if they did not have a confirmed CD or UC diagnosis, were an inpatient undergoing endoscopy or had incomplete data available.

### Ethical Approval

The study protocol was approved by the Western University Health Sciences Research Ethics Board (REB 117917). All methods were performed in accordance with the relevant guidelines and regulations of the Tri-Council Policy Statement.

### Study Outcomes

The objective of this study was to determine if more delays in endoscopy occurred during the period of the COVID-19 pandemic and if endoscopy delays in general and delays that occurred during the COVID-19 pandemic were associated with major adverse IBD events (defined as ER attendance, intestinal resection, hospitalization or escalation of drug therapy). Other objectives included characterizing the extent of delays (number and length) in endoscopy during the COVID-19 pandemic and if any IBD patient sub-groups based on age, sex or rurality were disproportionally impacted.

The primary endpoint was the occurrence of a major adverse IBD event after the date of their scheduled endoscopy where adverse outcomes included the following: presentation to the ER for an IBD-related complication including a suspected disease flare, an IBD medication-related adverse drug event or an extra-intestinal manifestation; hospitalization for an IBD-related complication including a suspected disease flare, an IBD medication-related adverse drug event or an extra-intestinal manifestation; an IBD-related surgery; or requiring an escalation in their IBD drug therapy, where escalation refers to an increase in the dose of a current IBD medication due to ongoing active disease or a change in drug therapy from 5-aminosalicylate to immunomodulator or biologic or from immunomodulator to biologic or the addition of rescue glucocorticoid therapy in addition to dose escalation. Secondary endpoints included each adverse outcome in singularity: IBD drug therapy escalation, surgery, ER attendance and hospitalization. Other endpoints included the number of endoscopies delayed in each cohort (2019 and 2020), and the median length of delay. Lastly, the effects of age, sex and rurality (defined based on postal code) on endoscopy delay were assessed. Participants were followed up to 6 months from the date of their scheduled endoscopy.

### Statistical Analysis

All statistical analyses were performed using GraphPad Prism 9 software (GraphPad Software Inc., San Diego, California). A *P*-value of <0.05 was considered significant. Descriptive statistics were used for demographic characteristics and are presented as medians with interquartile ranges (IQR) or means with standard deviations for continuous variables and frequency distributions with percentages for categorical variables. Differences between cohorts were assessed using a chi-squared test for categorical variables and the Mann–Whitney *U* test for continuous variables.

A chi-squared test was used to compare the proportion of patients experiencing any adverse IBD outcome to those not experiencing any adverse IBD outcome for participants where a delay in endoscopy occurred and where no endoscopy delay occurred. Additionally, adverse IBD outcomes by delay versus no delay were assessed for each of the cohorts (2019 and 2020) separately. For all other outcomes, a chi-squared test was used to compare proportions where appropriate.

Lastly, the effect of other covariates on the occurrence of endoscopy delay was assessed by multivariable logistic regression. The covariates assessed included age, sex, rurality, IBD disease type and time period of the delay. Data are presented as odds ratio (OR) with 95% confidence intervals (CI).

## RESULTS

**Figure 1.**
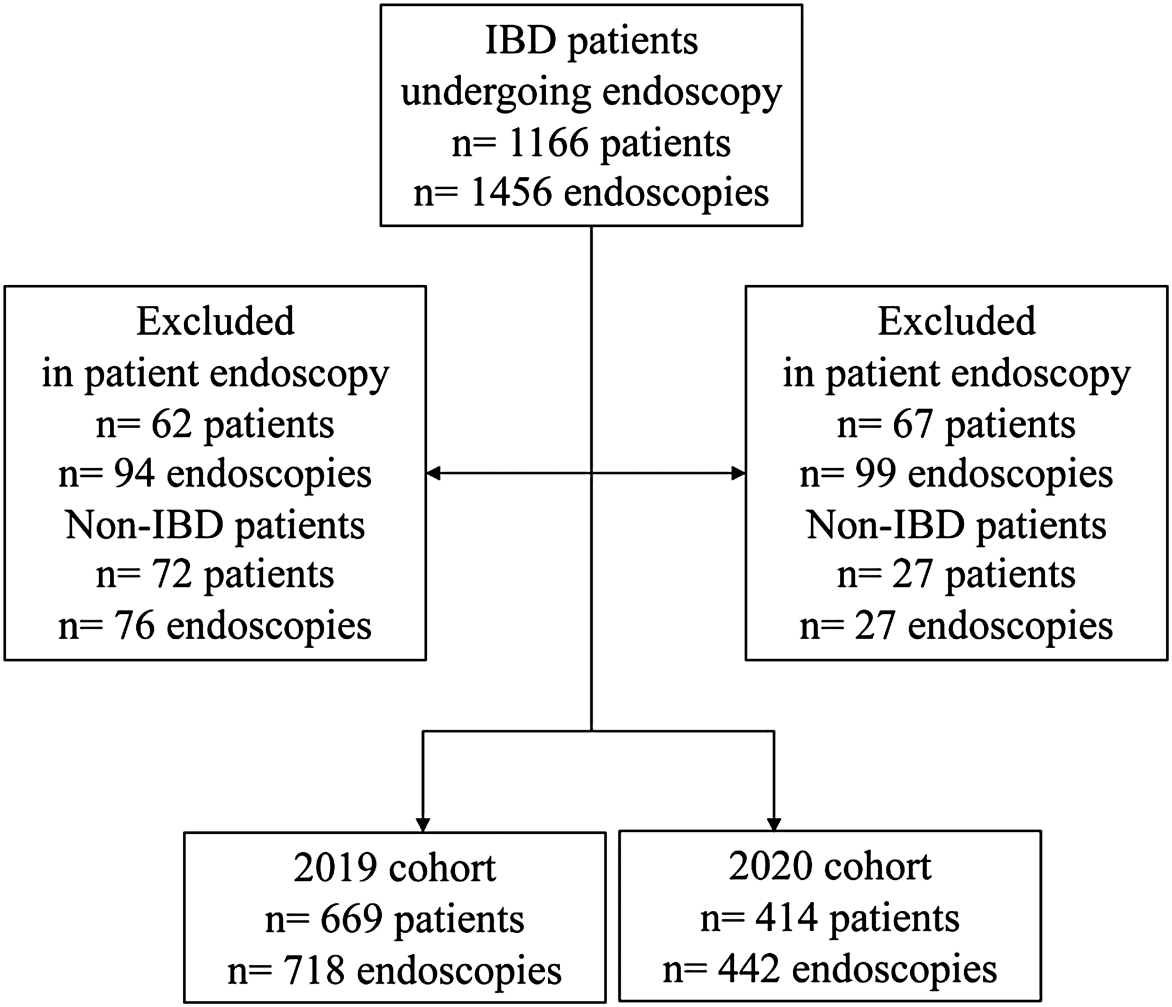
Study flow. IBD, Inflammatory bowel disease.

Participant selection is summarized in Figure 1. A total of 669 patients with IBD undergoing 718 endoscopies were included in the 2019 cohort (prior to the COVID-19 pandemic) and 414 patients with IBD undergoing 442 endoscopies were included in the 2020 cohort (during the COVID-19 pandemic). This was a 38% reduction in endoscopy overall from 2019 to 2020. Participants were additionally analyzed based on the occurrence of an endoscopy delay irrespective of the time period in which it occurred as well as by the time period of the delay. A total of 185 patients across 188 endoscopies experienced a delay in their procedure, whereas a total of 898 patients across 972 endoscopies were seen at their first scheduled endoscopy appointment. Demographic data presented by cohort year and by occurrence of delay versus no delay are summarized in Tables 1 and 2, respectively. No differences were seen in the baseline demographic characteristics based on cohort year or occurrence of endoscopy delay. The majority of patients presented for a colonoscopy and were from an urban centre. The indications for endoscopy in 2019 were as follows: surveillance for dysplasia (*n* = 68, 9.5%), symptoms suggestive of active IBD (*n* = 236, 32.9%), complications of IBD requiring endoscopic intervention (e.g., fibrotic stricture dilation) (*n* = 31, 4.3%), reassessment of disease activity post-treatment (*n* = 357, 49.7%) and evaluation for research study (*n* = 26, 3.6%). The indications for endoscopy in 2020 were as follows: surveillance for dysplasia (*n* = 19, 4.3%), symptoms suggestive of active IBD (*n* = 189, 42.8%), complications of IBD requiring endoscopic intervention (e.g., fibrotic stricture dilation) (*n* = 42, 9.5%), reassessment of disease activity post-treatment (*n* = 167, 37.8%) and evaluation for research study (*n* = 25, 5.6%).

**Table 1.** Baseline characteristics by cohort year

Patients	2019 (*n* = 669)	2020 (*n* = 414)	*P*-value
Mean age (SD)	46.7 (15.8)	47.0 (16.2)	0.84
CD diagnosis (%)	374 (56%)	240 (58%)	0.52
Female sex (%)	350 (52%)	221 (53%)	0.75
Rural status (%)	161 (24%)	100 (24%)	>0.99
Endoscopies	2019 (*n* = 718)	2020 (*n* = 442)	*P*-value
EGD	26 (3.6%)	27 (6.1%)	0.059
Colonoscopy	484 (67%)	280 (63%)	0.16
Sigmoidoscopy	113 (15.7%)	67 (15.2%)	0.80

CD, Crohn’s disease; n, number; SD, Standard deviation; EGD, esophagogastroduodenoscopy.

**Table 2.** Baseline cohort characteristics by delay

Patients	Delayed (*n* = 185)	Non-delayed (*n* = 898)	*P*-value
Mean age (SD)	48.2 (16.0)	46.6 (15.9)	0.24
CD diagnosis (%)	102 (55.1%)	512 (57.0%)	0.64
Female sex (%)	93 (50.3%)	478 (53.2%)	0.46
Rural status (%)	46 (24.9%)	215 (23.9%)	0.79
Endoscopies	Delayed (*n* = 188)	Non-delayed (*n* = 972)	*P*-value
EGD	7 (3.7%)	46 (4.7%)	0.54
Colonoscopy	132 (70.2%)	632 (65.0%)	0.17
Sigmoidoscopy	27 (14.4%)	153 (15.7%)	0.63

CD, Crohn’s disease; SD, Standard deviation; EGD, esophagogastroduodenoscopy.

A greater proportion of endoscopies were delayed during the COVID-19 pandemic (2019, *n* = 70/718, 9.7% vs. 2020, *n* = 115/442, 26.0%, *P* < 0.0001). This remained significant when adjusting for the covariates age, sex, rurality and disease type (OR = 3.39, 95% CI: 2.46–4.72, adjusted *P* < 0.0001) (Table 3). Median delay length was 56 days (IQR = 63) in 2020, during the pandemic, and 30 days (IQR = 42) in 2019, prior to the pandemic. The covariates age, sex, rurality and disease type were not associated with an increased risk of endoscopy delay (Table 3).

**Table 3.** Association between endoscopy delay and covariates

Variable	OR (95% CI)	*P*-value
Urban status	0.932 (0.647–1.36)	0.71
Female Sex	1.18 (0.853–1.62)	0.32
CD diagnosis	1.08 (0.778–1.49)	0.66
Age	1.01 (0.998–1.02)	0.13
Year of delay (2020)	**3.39 (2.46–4.72)**	**<0.0001**

OR, Odds ratio; CI, confidence interval; CD, Crohn’s disease.

Bold values indicate variables of statistical significance.

Additionally, the proportion of delayed endoscopies were assessed by indication and year. Indication reasons were combined into a ‘symptomatic endoscopy’ group versus an ‘asymptomatic endoscopy’ group whereby the symptomatic group included endoscopies performed for symptoms suggestive of active IBD and where endoscopic intervention for an IBD complication was warranted. The asymptomatic group included endoscopies performed for dysplasia surveillance, disease re-assessment post-treatment or for participation in a research study. In 2019, 9.5% of ‘symptomatic’ and 10.0% of ‘asymptomatic’ procedures were delayed respectively (*P* = 0.90). In 2020, 22.9% of ‘symptomatic’ and 30.8% of ‘asymptomatic’ procedures were delayed respectively (*P* = 0.06).

**Figure 2.**
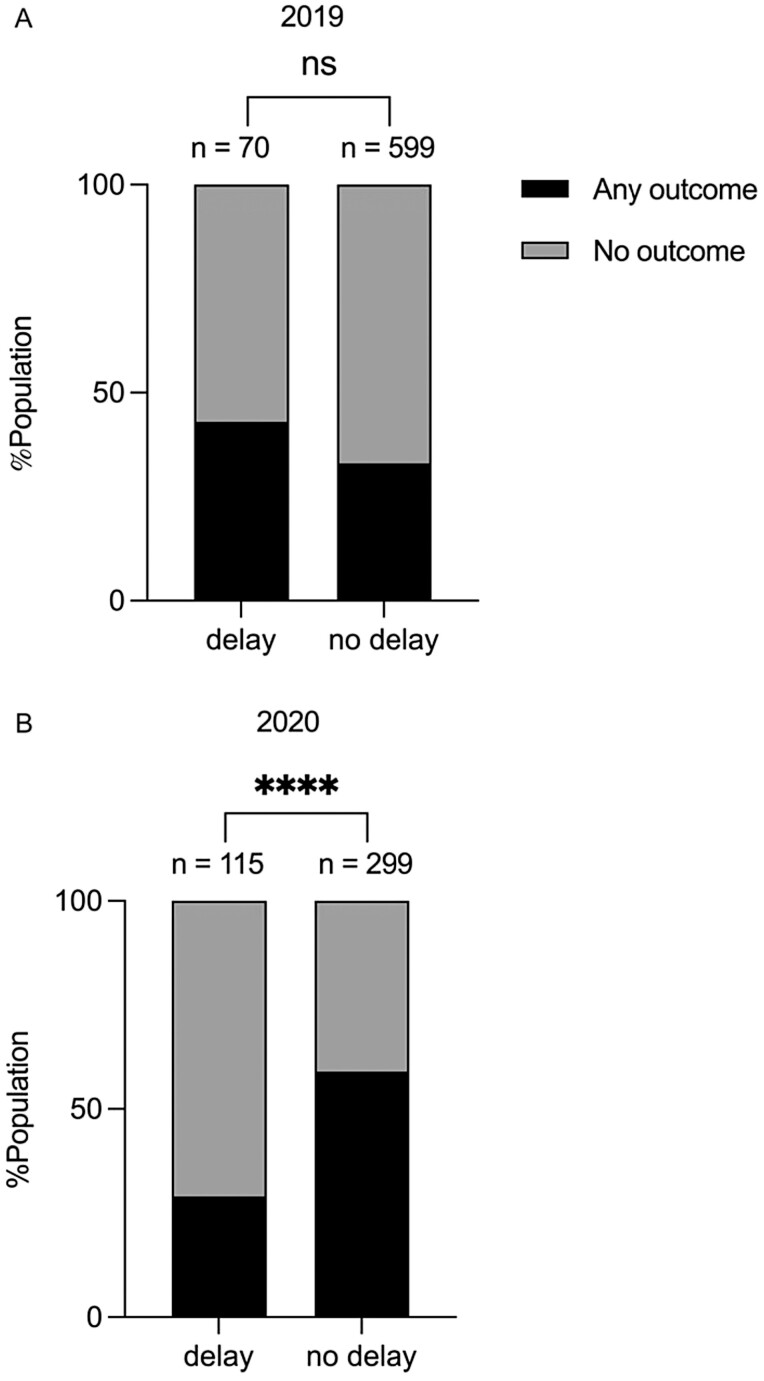
Percentage of IBD patients experiencing any major adverse IBD event by delay status in 2019 (A) and 2020 (B). Major adverse IBD events include hospitalization, surgery, escalation in drug therapy, or an ER visit. ns, not significant, ∗∗∗∗*P* < 0.0001; IBD, inflammatory bowel disease; %, percentage; ER, emergency room.

No difference was seen in the proportion of any major IBD adverse outcome in participants who sustained a delay in endoscopy versus those who did not (adverse IBD outcome: delayed, *n* = 63/185, 34.0% vs. no delay, *n* = 321/898, 35.7%, *P* = 0.66). After adjustment for baseline covariates, delay was not an independent predictor of the risk of a major adverse IBD outcome (Table 4). The proportion of delayed patients experiencing any adverse IBD outcome was assessed separately for each cohort (2019 vs. 2020). There was no difference in adverse IBD outcomes between the delayed and non-delayed groups in 2019 while in 2020, a higher proportion of adverse IBD outcomes occurred in the non-delayed cohort (*P* < 0.0001, Figure 2). The length of delay was not associated with an increased risk of adverse IBD outcome (OR = 0.99, 95% CI = 0.98–1.00, adjusted *P* = 0.11) when adjusting for sex, age, disease type and rurality.

**Table 4.** Association between covariates and the composite of major adverse IBD events

Variable	OR (95% CI)	*P*-value
Urban	**0.65 (0.48-87)**	**0.0043**
Female Sex	1.26 (0.99-1.60)	0.063
CD diagnosis	0.95 (0.75-1.22)	0.70
Age	1.00 (0.99-1.01)	0.78
Endoscopy delayed	1.23 (0.89-1.34)	0.20

OR, Odds ratio; CI, confidence interval; CD, Crohn’s disease.

Bold values indicate variables of statistical significance.

When the components of the major adverse IBD outcomes were analyzed separately, there was no difference in the secondary outcomes of ER attendance, hospitalization, surgical resection or escalation of drug therapy between participants who sustained a delay in endoscopy versus those who did not (Supplementary Table 1). This finding persisted even when assessed by delay and by year (Supplementary Table 2).

## DISCUSSION

The COVID-19 pandemic was associated with rapid, intensive and wide-scale changes to the provision of care to patients across a wide number disciplines in the hope of limiting the spread of the SARS-CoV2 virus (9). Such changes significantly affected procedurally based medical specialties, such as gastroenterology, where more delays in endoscopy were recommended (8,9). While the need for infection control measures is imperative during a pandemic, understanding the consequences of changes to care provision for patients, beyond those directly infected by the SARS-CoV2 virus, is essential for fully defining the impact of the COVID-19 pandemic. Such understanding allows for the adaptation of our community COVID-19 response, including how care is provided to all patients beyond those with SARS-CoV2, to ensure the best possible patient care.

In this study, we aimed to quantify if the COVID-19 pandemic was associated with key delays in endoscopy care in addition to how delays impacted IBD patients using objective measures. We found that there were significantly more endoscopy delays for patients with IBD assessed during the period of the pandemic than those seen prior to the pandemic, and that these delays were longer. This has been observed at other centres (10–12). Interestingly, sex, age, rurality and procedure type were not associated with having an endoscopic procedure delayed, highlighting an absence of disparity in procedural delay between individuals. Of course, it must be recognized that the usual delays in IBD care occurring pre-pandemic delays were likely dealt with differently and occurred for wholly different reasons than delays taking place during the pandemic.

Additionally, we saw an overall 38% reduction in the performance of any endoscopy for IBD patients between the 2019 and 2020 cohorts. This may be related to patient-driven factors associated with fear of contracting COVID-19 versus system-driven factors associated with non-urgent endoscopy reductions to minimize the spread of COVID-19. Longer term studies are needed to fully evaluate the impact of the pandemic on IBD endoscopy and the resultant effect on IBD outcomes.

Moreover, it should be noted that the outcome of treatment escalation had the potential to be biased towards patients who attended endoscopy; however, a similar incidence of treatment escalation was seen in the delayed and non-delayed groups when the 2019 and 2020 cohorts are considered together (16.2% vs. 17.8%). This similarity may be due to patients still having the opportunity to interact with their provider via other means (virtual, telephone) and having therapy changed based on non-endoscopic parameters such as clinical symptoms, biochemistries or stool biomarkers. Conversely, in 2020, fewer total adverse IBD outcomes occurred in the delayed population. One could infer that sicker patients were appropriately triaged to complete an endoscopy while less sick patients were delayed.

We also found that for the composite primary endpoint (any adverse event) in addition to the secondary endpoints (drug escalation, surgery, ER visit or hospitalization), there were no significant differences in the occurrence of these outcomes between the delayed and non-delayed cohorts. This opposes what has been previously published on delays in IBD diagnosis, including endoscopic diagnosis and disease outcomes. Schoepfer *et al.* reported more frequent intestinal stenosis and surgical intervention for patients with a delayed CD diagnosis, and Obi *et al.* demonstrated that delays in endoscopic assessment for hospitalized UC patients were associated with higher mortality, prolonged hospitalization, higher hospital costs and more frequent colectomy (13,14). It should be noted that the study completed by Schoepfer et al. evaluated individuals at first presentation of CD and this population was not included in the present study. They also assessed delays where the median length of delay was much longer (9 vs. 2 months) than what was reported in our study. Moreover, endoscopy delays during the COVID-19 pandemic (2020 cohort) were associated with fewer total deleterious IBD outcomes, suggesting that patients with IBD in need of urgent endoscopy were appropriately identified. There was a trend toward a greater proportion of ‘asymptomatic’ procedures being delayed in 2020; however, this did not achieve statistical significance. Interestingly, patients from an urban setting were significantly less likely to have any deleterious outcome compared to rural patients (Table 4). This has been seen in other Canadian IBD populations, where rural patients had less physician visits, more ER visits and hospitalizations (15).

Furthermore, it is important to recognize that the usual delays related to IBD care prior to the pandemic are most likely occurring for a different reason than delays occurring during the pandemic.

Limitations of this study include its retrospective study design, the absence of baseline disease severity data and its limited timeframe that did not encompass additional ‘waves’ of the COVID-19 pandemic. Despite these limitations, its robust sample size allows for a more accurate evaluation of the study’s outcomes.

## CONCLUSION

Ultimately, the COVID-19 pandemic was associated with more and longer delays in endoscopy for patients with IBD. In general, short-term delays were not associated with more deleterious immediate IBD outcomes. In fact, short-term delays seen during the pandemic were associated with fewer short-term adverse IBD outcomes. This suggests that patients with IBD were appropriately triaged to endoscopy during the pandemic, allowing earlier intervention and the prevention of adverse IBD outcomes. Additionally, this may reflect that patients who were triaged to attend endoscopy were sicker and more in need of care. Further studies are needed to assess the impact of longer endoscopy delays that may have been seen as populations experienced more ‘waves’ of COVID-19. Studies evaluating longer term IBD outcomes, such as the occurrence of fibrostenotic disease, are also needed.

## Supplementary Material

gwac009_suppl_Supplementary_Material
