# Supplementary material for: Short-Term COVID-19 Pandemic-Related Endoscopy Delays Did Not Translate to Deleterious Outcomes for Patients With Inflammatory Bowel Disease: A Retrospective Cohort Study
Source: J Can Assoc Gastroenterol. 2022 Mar 16;5(6):251–5. doi: 10.1093/jcag/gwac009 (PMC8992274; doi:10.1093/jcag/gwac009)
Supplement: gwac009_suppl_Supplementary_Material [file gwac009_suppl_supplementary_material.docx]

**Supplementary Material: Short-term COVID-19 pandemic-related endoscopy delays did not translate to deleterious outcomes for patients with inflammatory bowel disease: a retrospective cohort study**

Supplementary Table 1. Any major adverse IBD outcomes by endoscopy delay status

| **Outcome** | **Delay (n=185)** | **No Delay (n=898)** | **p-value** |
| --- | --- | --- | --- |
| ER attendance | 30 (16.2) | 165 (18.4) | 0.49 |
| Hospitalization | 13 (7.0) | 70 (7.8) | 0.72 |
| Surgical resection | 4 (2.2) | 22 (2.4) | 0.82 |
| Treatment escalation | 30 (16.2) | 160 (17.8) | 0.60 |

Inflammatory bowel disease, IBD; Emergency room, ER

Supplementary Table 2. Major adverse IBD outcomes by endoscopy delay status and year

| **Adverse Outcome** | **2019 (n=669)** | | | **2020 (n=414)** | | |
| --- | --- | --- | --- | --- | --- | --- |
|  | **Delay (n=70)** | **No delay (n=599)** | **p-value** | **Delay (n=115)** | **No delay (n=299)** | **p-value** |
| Surgical resection | 2/70 (2.8%) | 13/599 (2.2%) | 0.71 | 2/115 (1.7%) | 9/299 (3.0%) | 0.73 |
| ER attendance | 17/70 (24.3%) | 107/599 (17.9%) | 0.19 | 13/115 (11.3%) | 58/299 (19.4%) | 0.06 |
| Hospitalization | 5/70 (7.1%) | 34/599 (5.7%) | 0.62 | 8/115 (7.0%) | 36/299 (12.0%) | 0.16 |
| Treatment escalation | 13/70 (18.6%) | 98/599 (16.4%) | 0.64 | 17/115 (14.8%) | 62/299 (20.7%) | 0.21 |

Inflammatory bowel disease, IBD; Emergency room, ER
